# Supplementary figures and images for: Purification and characterization of detergent stable alkaline lipase from Bacillus safensis TKW3 isolated from Tso Kar brackish water lake
Source: PeerJ. 2025 Feb 19;13:e18921. doi: 10.7717/peerj.18921 (PMC11846503; doi:10.7717/peerj.18921)

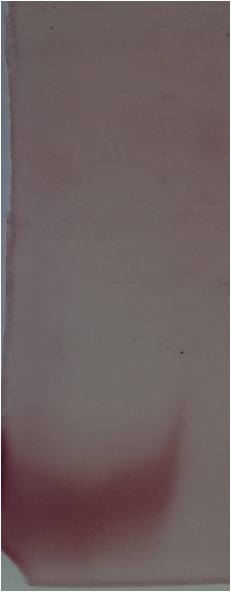

Supplement: Supplemental Information 1 — SDS-PAGE and Native PAGE Analysis of Lipase Activity and Molecular Weight Determination [file peerj-13-18921-s001.zip › Zymogram.jpg]

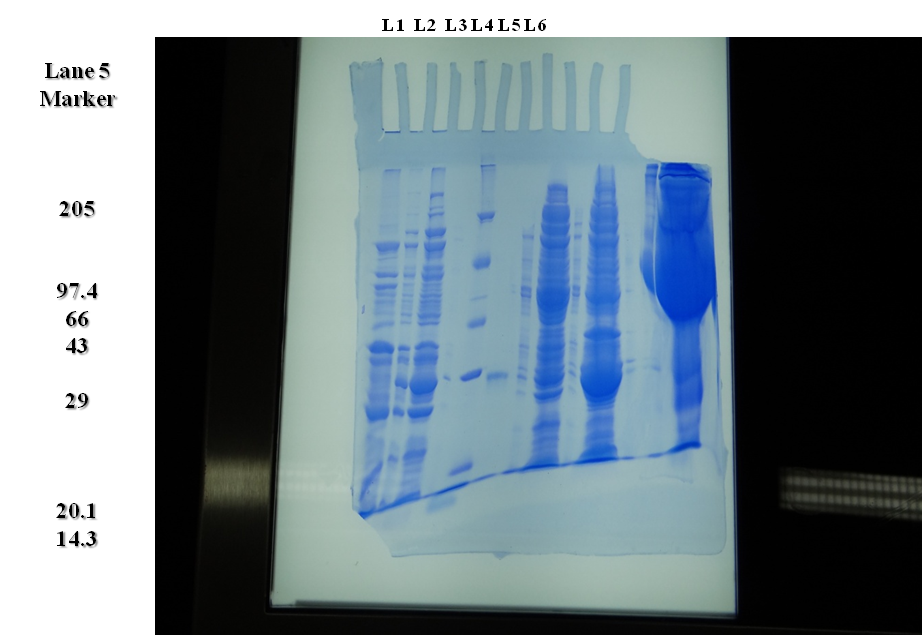

Supplement: Supplemental Information 1 — SDS-PAGE and Native PAGE Analysis of Lipase Activity and Molecular Weight Determination [file peerj-13-18921-s001.zip › Picture1 SDS Page.png]

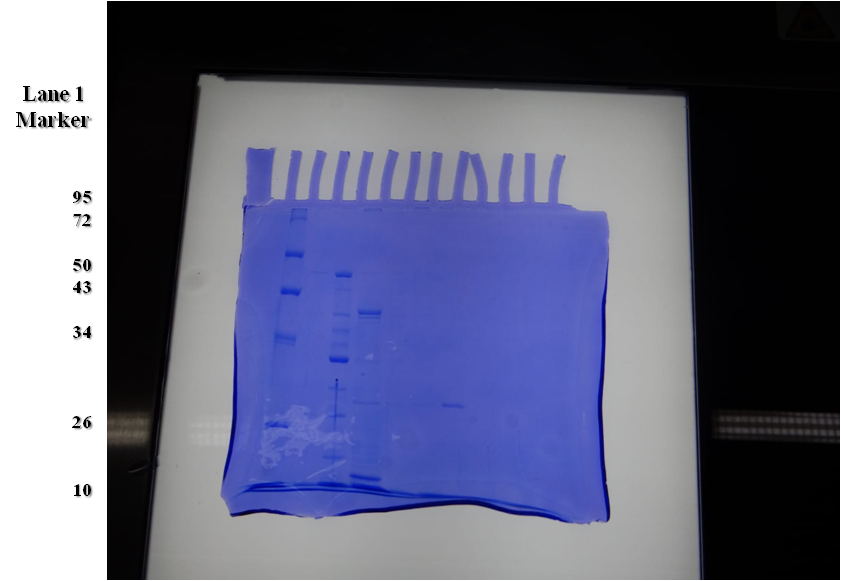

Supplement: Supplemental Information 1 — SDS-PAGE and Native PAGE Analysis of Lipase Activity and Molecular Weight Determination [file peerj-13-18921-s001.zip › Picture2 SDS.png]

# Figure 1

Standard curve of pN

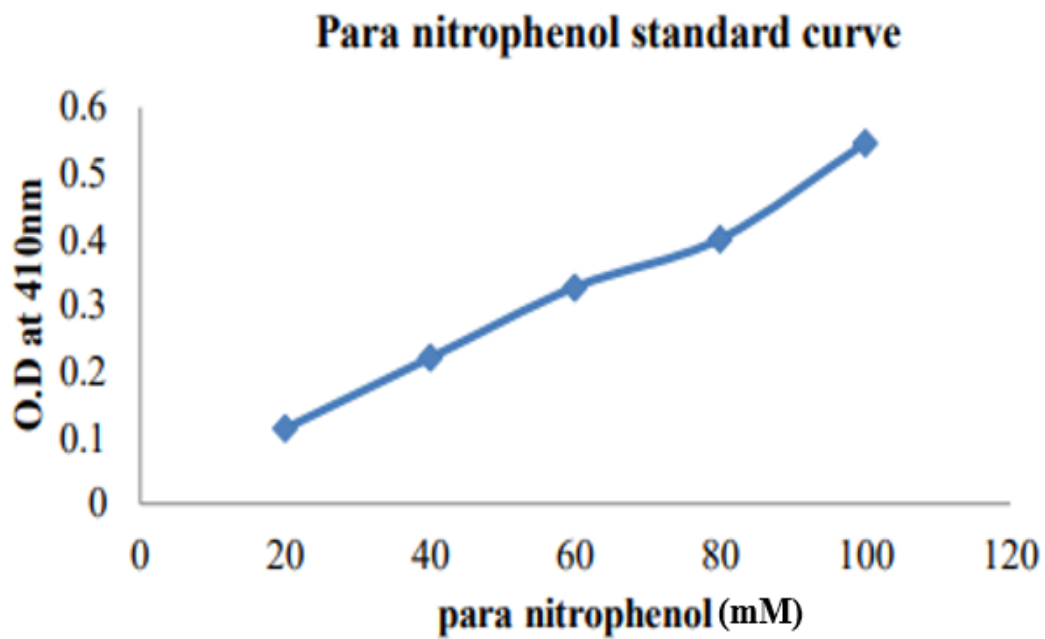

Standard curve of different concentrations of pNP at 410 nm

Supplement: Supplemental Information 2 [file peerj-13-18921-s002.pdf]
