# Supplementary material for: Purification and characterization of detergent stable alkaline lipase from Bacillus safensis TKW3 isolated from Tso Kar brackish water lake
Source: PeerJ. 2025 Feb 19;13:e18921. doi: 10.7717/peerj.18921 (PMC11846503; doi:10.7717/peerj.18921)
Supplement: Supplemental Information 3 — The phylogenetic tree was constructed using the MEGA 4.1 program. [file peerj-13-18921-s003.pdf]

Figure 2

Phylogenetic tree of TKW3 strain.

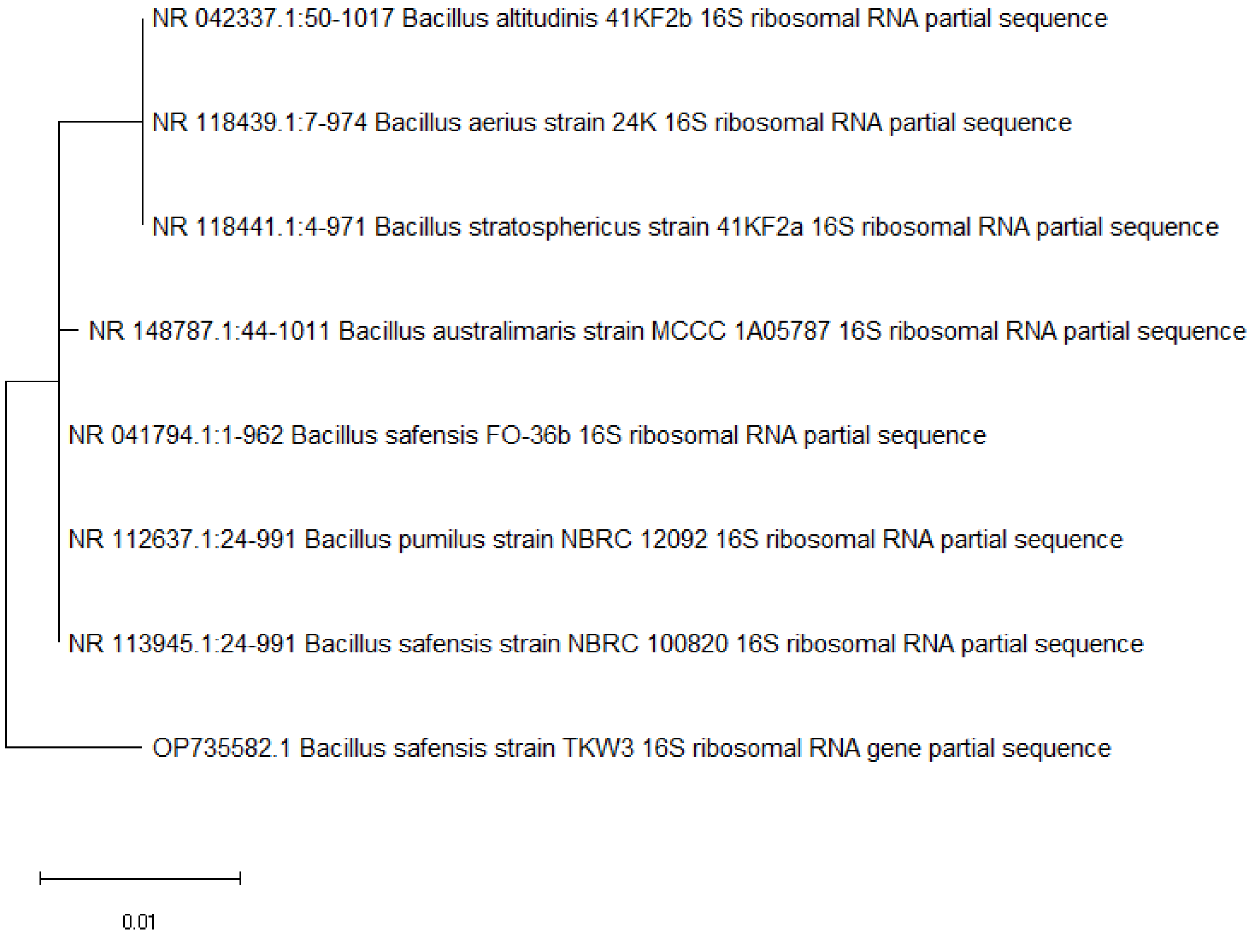

Phylogenetic tree of TKW3 strain. The phylogenetic tree was constructed using the MEGA 4.1 program
