# Supplementary material for: Purification and characterization of detergent stable alkaline lipase from Bacillus safensis TKW3 isolated from Tso Kar brackish water lake
Source: PeerJ. 2025 Feb 19;13:e18921. doi: 10.7717/peerj.18921 (PMC11846503; doi:10.7717/peerj.18921)

Figure 3

Secondary screening of TKW3 strain on Tributyrin substrate plate to check the localization of lipase enzyme. TKW3 showed intracellular enzyme activity on Agar plates supplemented with Tributyrin as substrate.

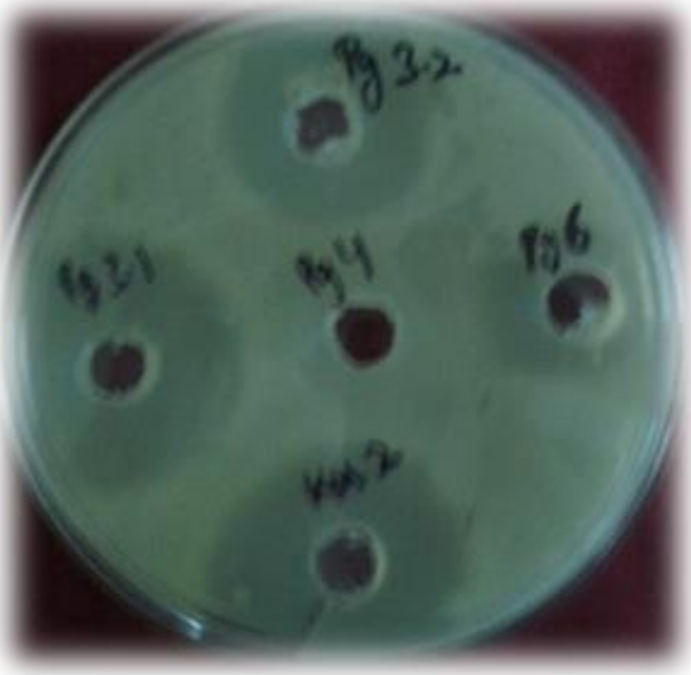

Supplement: Supplemental Information 4 — TKW3 showed intracellular enzyme activity on Agar plates supplemented with Tributyrin as substrate. [file peerj-13-18921-s004.pdf]
