# Supplementary material for: Purification and characterization of detergent stable alkaline lipase from Bacillus safensis TKW3 isolated from Tso Kar brackish water lake
Source: PeerJ. 2025 Feb 19;13:e18921. doi: 10.7717/peerj.18921 (PMC11846503; doi:10.7717/peerj.18921)
Supplement: Supplemental Information 5 [file peerj-13-18921-s005.pdf]

Temperature optima		1	2	3	Mean
	5°c	51.91	52.65	52.89	52.48
	10°c	75.1	74.79	74.22	74.7
	15°c	77.69	77.88	77.95	77.84
	20°c	81.62	82.43	81.8	81.95
	25°c	94.68	94.51	94.88	94.69
	30°c	117.26	117.37	117.09	117.24
	37°c	111.24	111.42	111.39	111.35
	45°c	98.72	98.54	98.81	98.69
	50°c	93.8	93.71	93.62	93.71
	55°c	66.38	66.73	66.57	66.56
	60°c	60.61	60.48	60.88	60.656
	65°c	53.38	53.22	53.31	53.303
	70°c	51.65	51.87	51.35	51.62
	75°c	50.58	50.24	49.99	50.27
	80°c	39.72	40.16	40.03	39.97

pH optima		1	2	3	Mean
	4	0.1711	0.1719	0.1728	0.1719
	5	0.496	0.479	0.489	0.488
	6	1.371	1.377	1.398	1.382
	7	63.26	63.4	63.63	63.43
	8	82.98	82.93	82.79	82.9
	9	98.31	98.47	98.27	98.35
	10	68.941	68.88	68.78	68.867
	11	31.48	31.75	31.63	31.62
	12	15.37	15.689	15.45	15.503

Effect of NaCl on the activity		1	2	3	Mean
	0.5M	23.71	23.87	23.94	23.84
	1M	63.847	63.798	63.89	63.845
	2M	91.511	91.39	91.431	91.444
	3M	141.218	141.222	141.229	141.223
	4M	129.02	128.97	129.1	129.03
	5M	110.1	109.92	109.98	110
	6M	15.05	15.17	15.08	15.1

Effect of inhibitors		2mM			
		1	2	3	Mean
	Control	54.33	54.07	54.2	54.2
	EDTA	83.99	83.81	84.38	84.06
	β-ME	3.19	3.55	3.76	3.5
	DDt	5.77	4.84	5.29	5.3
	Gn-HCl	52.91	53.28	53.11	53.1

Effect of Metal ions		1	2	3	Mean
	**Control**	58.19	57.27	58.36	57.94
	**Ca2+**	58.34	59.28	58.39	58.67
	**Mg2+**	60.07	59.81	60.01	59.96333
	**Cu2+**	14.7	15.1	15.3	15.03333
	**Li+**	61.5	61.47	61.56	61.51
	**K+**	60.27	60.31	60.38	60.32

	Substrates specificity	1	2	3	Mean
	C2	10.4	10.38	10.48	10.4
	C4	31.8	31.87	31.85	31.84
	C6	50.31	50.38	50.42	50.37
	C8	57.88	57.9	57.95	57.9
	C10	42.11	42.38	42.29	42.26
	C12	28.31	28.37	28.19	28.37
	C14	8.19	7.99	8.13	8.1

Detergent compatibility		(Control)			
		1	2	3	Mean
	5°C	141.4	140.7	140.9	141
	**10°C**	151.1	151.3	150.6	151
	**20°C**	182.9	183.2	183.6	183.2
	**30°C**	190.4	189.1	190.7	190
	**37°C**	137.1	137.5	136.7	137.1
	**45°C**	101.8	102.5	102.1	102.1

		Temperature stability		1	2
			**5°c**	106.84	106.67
			**10°c**	106.05	106.36
			**15°c**	105.67	105.43
			**20°c**	105.98	105.64
			**25°c**	104.88	105.04
			**30°c**	104.81	104.93
			**37°c**	104.35	104.03
			**45°c**	99.24	99.11
			**50°c**	92.4	92.37
			**55°c**	86.79	87.04
			**60°c**	50.76	50.55
			**65°c**	49.04	48.98
			**70°c**	47.41	47.75
			**75°c**	48.13	47.91
			**80°c**	42.87	43.14

		pH stability		1	2
			**4**	0.0019	0.0023
			**5**	0.092	0.102
			**6**	0.901	0.905
			**7**	93.75	93.81
			**8**	102.37	102.49
			**9**	99.47	99.69
			**10**	53.38	53.46
			**11**	13.3	12.98
			**12**	0.995	1.078

		Effect of NaCl on the stability.		1	2
			**0.5M**	91.98	91.81
			**1M**	93.21	93.25
			**2M**	130.72	130.91
			**3M**	146.2	146.11
			**4M**	108.67	108.61
			**5M**	95.1	95.01
			**6M**	82.89	82.81

		5mM			
Relative activity		1	2	3	Mean
100		55.89	55.58	55.63	55.7
155.0922509		98.44	98.11	98.65	98.4
6.457564576		1.02	1.009	1.07	1.03
9.778597786		3.53	3.41	3.86	3.6
97.9704797		49.651	49.99	50.24	49.96

**Relative activity**
100
101.2599241
103.4921183
25.94638131
106.1615464
104.1076976

**Relative activity**
18
55
87
100
73
49
14

	**Tide**				
	**1**	**2**	**3**	**Mean**	
	134.7	134.99	135.41	135	
	149.12	149.3	148.72	149	
	167.53	168.33	168.2	168	
	211.74	212.05	212.23	212	
	131.1	131.07	130.84	131	
	96.98	96.73	97.29	97	

3	Mean	Relative activity
107.22	106.91	100
106.49	106.3	99.42942662
105.1	105.4	98.58759704
105.48	105.7	98.8682069
105.38	105.1	98.30698719
104.99	104.91	98.12926761
103.92	104.1	97.37162099
99.43	99.26	92.8444486
92.28	92.35	86.38106819
86.87	86.9	81.28332242
50.88	50.73	47.45112712
48.71	48.91	45.74876064
47.52	47.56	44.48601628
47.981	48.007	44.90412496
43.08	43.03	40.24880741

3	Mean	Relative activity
0.0021	0.0021	0.00204918
0.106	0.1	0.097580016
0.9	0.902	0.880171741
93.84	93.8	91.53005464
102.58	102.48	100
99.76	99.64	97.22872756
53.6	53.48	52.18579235
12.78	13.02	12.70491803
1.14	1.071	1.045081967

3	Mean	Relative activity
91.91	91.9	62.85909713
93.32	93.26	63.78932969
130.86	130.83	89.4870041
146.29	146.2	100
108.52	108.6	74.28180575
95.07	95.06	65.02051984
82.7	82.8	56.63474692

Relative activity
100
176.6606822
1.849192101
6.463195691
89.69511848

C2
C4
C6
C8
C10
C12
C14

Ariel					
**Mean**	**1**	**2**	**3**	**Mean**	
135	143.3	143.02	142.7	143	
149	143.89	144.14	143.99	144	
168	166.11	165.91	165.69	165.9	
212	146.98	147.53	147.09	147.2	
131	101.73	102.38	101.61	101.9	
97	75.7	76.01	76.31	76	

%

18
55
87
100
73
49
14

Surf excel			
**1**	**2**	**3**	**Mean**
153.58	153.72	152.91	153.4
159.71	160.23	160.11	160
200.61	200.17	200.42	200.4
230.03	230.32	229.73	230
162.3	163.31	163.1	162.9
120.72	120.7	120.39	120.6
