# Supplementary material for: Purification and characterization of detergent stable alkaline lipase from Bacillus safensis TKW3 isolated from Tso Kar brackish water lake
Source: PeerJ. 2025 Feb 19;13:e18921. doi: 10.7717/peerj.18921 (PMC11846503; doi:10.7717/peerj.18921)
Supplement: Supplemental Information 6 [file peerj-13-18921-s006.docx]

**Screening of carboxylic ester hydrolases from the Tso Kar brackish water lake using a culture-dependent approach**

**1. Material and Methods**

**1.1 Enrichment and isolation of halophiles**

The isolation of halophiles from the Tso Kar water sample was done by salt enrichment (Khunt and Pandhi, 2011). The collected sample was enriched by periodic sub-culturing of samples in halophilic media composed of casein acid hydrolysate-10 (g/l), protease peptone- 5(g/l), potassium chloride- 2 (g/l), yeast extract- 10 (g/l), trisodium citrate- 3 (g/l), magnesium sulfate-25 (g/l), sodium chloride- 50 to 410 (g/l), pH of medium was adjusted to 7.0 to 7.4 (Khunt and Pandhi, 2011). A serial dilution technique was used to isolate the bacterial strains. The water sample was serially diluted. Different dilutions of the water sample were spread plated on basal media enriched with salt and incubated at 37 ̊C for up to 72 hrs. Resultant colonies from enriched 12% NaCl halophilic media were purified by repeated streaking for the isolation of pure cultures on respective agar media and stored at 4 ºC.

The isolates were streaked on the halophilic media (pH 7.2) and salt concentration 12% w/v). The plates containing pure cultures were stored at 4 °C. The microorganisms were subcultured monthly. All 15 purified isolates were also preserved in glycerol stock cultures and stored at -80 °C.

**1.2 Screening of halophilic isolates for esterase/lipase production**

The isolated halophilic isolates were screened by a sensitive and specific plate assay on basal medium containing 1% tributyrin for esterase and 1% olive oil for lipase enzyme activity.

**1.2.1 Primary screening for carboxylic ester hydrolases**

Primary screening of the halophilic isolates for esterase/lipase enzyme activity was done on solid media containing Tributyrin and olive oil as substrates. Lipolysis was observed after 48 hrs of incubation at 37 °C directly by changes in the appearance of the substrates, which were emulsified mechanically in growth media and poured into a petridish. Isolates that showed clear zones of Tributyrin hydrolysis were identified as esterase producers while the screening of lipase activity was monitored on Rhodamine agar plates containing 1% olive oil. The hydrolysis of substrate olive oil causes the formation of orange fluorescent halos around the bacterial colonies or presence of orange fluorescent colonies present on the plates observed under UV irradiation. Potent enzyme producers were chosen and screened further to determine the localization of the enzyme.

**1.2.1 Secondary screening for carboxylic ester hydrolases**

Isolated halophilic strains were then screened for the localization of enzyme activity. The isolates were inoculated in an enriched halophilic broth for enzyme production. Inoculated broths were then incubated at 30 °C on an incubator shaker at 181 rpm. After 48 hrs of incubation, the grown cultures were centrifuged at 10,000 g for 30 minutes to collect the supernatant and pellet. The supernatant of the isolates were used as the fractions having extracellular enzyme. Cell pellets were resuspended in 50 mM phosphate buffer (pH 7), followed by disruption of cells by ultrasonication at 4 °C using a Q-Sonica sonicator. There are a total of five cycles, each of which consists of a one-minute pulse followed by 4 minutes pause at 15 kHz. The disrupted cell suspensions were then centrifuged at 10,000 g for about 30 min at 4 °C to collect the supernatant (CFE) having intracellular fractions of the enzyme. Both extracellular and intracellular enzyme fractions were analyzed for enzyme activity.

**1.3 Morphological Identification of Potential Halophilic Isolates**

Morphological identification of potential isolates TKW1, TKW3, and TKW12 was done by considering both macroscopic and microscopic characters.

**1.3.1 Colony-based macroscopic characterization**

The pure cultures of the selected isolate bacteria were streaked on the agar plate of halophilic media with corresponding enrichment conditions of 12% NaCl w/v and pH 7.5, and their colony characteristics in terms of shape, margin, and texture were observed for primary characterization.

**1.3.2 Cell morphology**

The selected strains, designated as TKW1, TKW3, and TKW12, were subjected to Gram staining for microscopic characterization in order to differentiate the bacteria on the basis of their cell morphology and cell arrangement. Gram staining, also called Gram's Method, is a method of differentiating bacterial species into two large groups: gram-positive and gram-negative. The name comes from the Danish bacteriologist Hans Christian Gram, who developed the technique. Gram staining was performed using a Gram staining kit (Himedia) following the manufacturer’s guidelines.

**Results**

**1 Isolation of halophiles**

Halophiles from the water sample of Tso Kar brackish lake in the Ladakh region were explored using a culture-dependent approach. In hypersaline environments such as lakes or salterns, extreme halophiles constitute a group of microorganisms that are well represented (OREN, 2002). Most of the research on halophilic organisms has been done on halophiles isolated from soda lake (Guadie et al., 2018), solar salterns (Lee et al., 2016), deep sea hydrothermal vents (Kaye et al., 2011), saline lakes (Vahed et al., 2018), ocean seas (Gaboyer et al., 2014), saline and alkaline soils (Gan et al., 2018), (Lee et al., 2015), salty food (Oguntoyinbo et al., 2018), etc.

A total of fifteen isolates from the water sample were isolated and purified by repeated streaking. The strains were maintained by repeated sub culturing on halophilic media having 12% salt concentration. In extreme environments, bacterial diversity is typically quite low. Four salt tolerant bacterial strains were isolated from soil samples collected from Shatkhira, Bangladesh (Mohanta et al., 2020). All of the isolated strains from Tso Kar water were able to thrive well at 12% NaCl concentration and tolerate salinity. It was found that halophilic isolates from several Indian sun salterns can tolerate salt and thrive in culture conditions with 15% sodium chloride (NaCl) (Radhakrishnan and Gopalan, 2022b).

**2 Screening of halophiles for carboxylic ester hydrolases**

All the purified isolates were then screened for the carboxylic ester hydrolase enzymes, specifically esterase and lipase, using tributyrin and olive oil as substrates. The isolates having zones of hydrolysis on tributyrin agar plates were esterase producers, and the isolates forming orange halos on Rhodamine B olive oil agar plates were regarded as lipase producers. Screening using Tributyrin agar plate assay, Rhodamine B olive oil agar plate assay, and Tween-20/Tween-80 hydrolysis was reported for esterase and lipase (Kumar et al., 2012). Carboxylesterase and lipase production of isolates was detected on media containing different substrates (Tchigvintsev et al., 2015). The screening of lipase producing microorganisms was reported using a 22% Tween-80 substrate (Kanlayakrit and Boonpan, 2007). Lipase producing organism were also screened on a medium having 2.5% olive oil and 0.001% rhodamine B and positive organism can be identified as an orange fluorescent halos under UV light (Bhatnagar et al., 2005).

Three of the bacterial isolates, designated TKW1, TKW3, and TKW12, were selected on the basis of zone index for esterase/lipase production. TKW1 and TKW12 isolates were the prominent esterase producers, while TKW3 was the prominent lipase producer among the isolated strains from the Tso Kar water sample. *Alkalibacterium* sp. was the first reported esterase from a halophilic cold-adapted environment (Wang et al., 2016b). Prominent esterase producers have been reported from the Halomonas genus in different hypersaline environments in south Spain (Sánchez‐Porro et al., 2003)**.**

**3 Identification of potential halophilic isolates**

The bacterial isolates TKW1 and TKW12 were characterized as gram-negative and rod-shaped strains. It has been reported that most halophiles belong to the gram-negative group (Ventosa et al., 1998). *Halomonas saliphila*, isolated from a saline soil sample from Gansu Province, PR China (Gan et al., 2018), *H tabrizica,* isolated from Urmia Lake of Iran (Vahed et al., 2018), *Halomonas ventosae,* isolated from saline soils in south-eastern Spain (Martínez-Cánovas et al., 2004), *H. argentinensis* (Ihara et al., 1997), and *H. saccharovorum* (McGenity and Grant, 1995), etc. share the properties of being gram-negative and rod-shaped halophiles. The strain *Bacillus safensis* TKW3 was characterized as a gram-positive, rod-shaped bacterium. Similar properties have been shared by *B. safensis* strains isolated from spacecraft and assembly-facility surfaces (Satomi et al., 2006).
